# Effects of virtual reality technology on early mobility in critically ill adult patients: a systematic review and meta-analysis

**DOI:** 10.3389/fneur.2024.1469079

**Published:** 2025-02-05

**Authors:** Yansha He, Qin Yang, Xiaoxia Dai, Tian Chen, Huan Wu, Kunjie Li, Shiqiong Zhu, Yanlin Liu, Hua Lei

**Affiliations:** ^1^Department of Neurosurgery, Chongzhou People’s Hospital, Chengdu, Sichuan, China; ^2^Department of Rehabilitation Medicine Center, Sichuan Provincial People’s Hospital, University of Electronic Science and Technology of China, Chengdu, China; ^3^Department of ICU, Sichuan Provincial People’s Hospital, University of Electronic Science and Technology of China, Chengdu, China; ^4^School of Medicine, University of Electronic Science and Technology of China, Chengdu, China; ^5^Department of Administration, Chongzhou People’s Hospital, Chengdu, Sichuan, China

**Keywords:** virtual reality, critically ill patients, motor function, meta-analysis, systematic review

## Abstract

**Objective:**

The study aimed to explore the effects of virtual reality (VR) technology on motor function rehabilitation in critically ill patients.

**Methods:**

Adhering to the PRISMA systematic evaluation method for developing the literature ranking criteria and search strategy, the following databases were systematically searched: PubMed, Embase, Web of Science, the Cochrane Library, CNKI, Wanfang Data, Chinese Medical Journal Full-Text Database, and SINOMED. The search focused on the impact of virtual reality technology on limb function rehabilitation in critically ill patients, covering the period from the database’s inception to 6 December 2024. To evaluate the quality of the included studies, the risk of bias was assessed using the RevMan 5.4 tool.

**Results:**

A total of 11 randomized controlled trials (RCTs) published over 10 years were included, involving 880 critically ill adult patients: 435 in the experimental group and 445 in the control group. The meta-analysis showed that, compared to the control group, virtual reality technology significantly improved the Berg Balance Scale (BBS) score (MD = 3.95, 95% CI: 3.19, 4.70, *p* < 0.05), the Functional Independence Measure (FIM) score (MD = 0.21, 95% CI: −1.35, 1.76, *p* > 0.05), the Functional Ambulation Category (FAC) score (MD = 0.72, 95% CI: 0.49, 0.94, *p* < 0.05), the upper limb motor function (Fugl-Meyer Assessment, FMA) score (MD = 5.08, 95% CI: 3.46, 6.69, *p* < 0.05), and the lower limb motor function (Fugl-Meyer Assessment FMA) score (MD = 2.83, 95% CI: 1.99, 3.67, *p* < 0.05) of the adult critically ill patients.

**Conclusion:**

Compared to traditional rehabilitation techniques, virtual reality technology has a better overall effect in improving motor rehabilitation in critically ill patients and enhancing balance, functional walking, and upper and lower limb motor functions. However, the effect of enhancing the functional independence of limbs is not yet evident and still needs to be confirmed by high-quality, multicenter, and large-sample clinical trials.

**Systematic review registration:**

https://www.crd.york.ac.uk/PROSPERO/ Prospero register No.: CRD42024546409.

## 1 Introduction

Virtual Reality (VR) is a computer simulation system that immerses users in a virtual environment mirroring the real world (1). In 2016, the Chinese government introduced VR in its 13th Five-Year Plan (2), committing to support VR technology in the future. In 2018, the policy document “Guiding Opinions on Accelerating the Development of the Virtual Reality Industry” outlined specific guidelines and support measures for the application of virtual reality technology in healthcare (3). All of these initiatives indicate China’s emphasis on and support for the development of VR, along with its aspirations for the widespread application of VR in the medical industry. The 2023 policy document titled “Guiding Opinions on Innovative Development of Humanoid Robots” released by the Ministry of Industry and Information Technology (MIIT) in China mentions the need to strengthen the application of “5G + Virtual Reality (VR) technology” in immersive experience scenarios (4). It also calls for innovation in the development of interactive and personalized products and services (3). Furthermore, it indicates that China has further requirements for VR application to meet the demands of intelligence and customization.

Early mobility is defined as rehabilitation activities that begin within 72 h of admission to the intensive care unit (ICU) (5, 6), typically within 24–48 h after achieving hemodynamic and respiratory stability (6–8). The intensive care unit (ICU) is equipped with numerous instruments and devices that can complicate patient mobility and may create challenges in implementing early rehabilitation activities. In addition, critically ill patients with multiple tubes inserted into their bodies are unable to move independently and have restricted mobility. As clinical treatment advancements continue to improve, early functional recovery in critically ill patients has become a primary focus in critical care medicine (9).

Traditional rehabilitation exercises typically includes passive joint movement, limb massage, isometric muscle contractions, and balance function training, which require a significant amount of manpower. However, in the intensive care unit, patients are often in critical condition, and medical staff are busy with various responsibilities, making it difficult to ensure effective implementation of traditional rehabilitation training. Many of their tasks require the assistance of instruments, some of which are difficult to move, such as magnetic therapy devices and traction devices. ICU space may be limited, and instruments such as exoskeleton robots may not be fully functional. In addition, therapists can only spend a limited amount of time with each patient (such as 20 min for daily activities and assessments). Moreover, the process can be quite dull, resulting in low motivation and compliance from patients, which makes it difficult to keep them engaged over the long term. The early mobility of critically ill adult patients is usually limited to their hospital beds, with many even unable to sit or stand, with limited rehabilitation options available. VR can stimulate a patient’s senses and promote active movement. As an early intervention for post-intensive care syndrome (PICS) in the ICU and a treatment method aimed at improving patient outcomes, VR technology can enhance patients’ experience, relieve pain, increase neurocognition, promote better sleep, boost patients’ confidence in their recovery, support rehabilitation training, improve patients’ psychological wellbeing, enhance patients’ quality of life, and lead to better long-term social outcomes (10–12). Overwhelming evidence (10, 13–17) suggests that early mobility exercises for critically ill patients can mitigate muscle atrophy and facilitate the recovery of physical functions. VR technology offers a novel solution for the daily exercise rehabilitation of critically ill patients. Haghedooren E et al. (18) suggested that VR rehabilitation for critically ill patients in the ICU is safe, feasible, and appreciated by the majority of patients. VR, as a game-based approach that may change the rehabilitation of critically ill ICU patients, should encourage subsequent research and development in augmented reality, with a focus on neuromuscular and cognitive efficacy. However, the use of VR in the ICU remains challenging. According to Bruno et al. (13), users may experience side effects, such as “cybersickness,” which is influenced by scenes with motion. It has also been pointed out that VR tends to cause less motion sickness than augmented reality (AR) sessions. Furthermore, critically ill patients are among the most vulnerable patient groups and warrant special ethical considerations if VR is introduced into daily care. Existing original research (19–22) has shown varying effectiveness of VR in rehabilitating motor function in critically ill patients, with specific differences in research outcomes. This review systematically searched for relevant studies and employed a meta-analysis to explore whether VR technology is more effective than conventional rehabilitation techniques in the early mobilization of critically ill adult patients. It is crucial to provide evidence-based support to help clinical healthcare professionals decide whether to use VR for early mobilization activities in critically ill patients and to indirectly assess the cost-effectiveness ratio between VR and traditional rehabilitation methods.

## 2 Materials and methods

### 2.1 Study design

Quantitative studies examining the use of virtual reality technology for motor function exercises in critically ill bedridden patients were identified by searching various Chinese and English databases. A meta-analysis was conducted on different scales measuring motor function, including the Berg Balance Scale (BBS), the Functional Independence Measure (FIM), the Fugl-Meyer Assessment (FMA), and the Functional Ambulation Category (FAC). The goal of this analysis is to understand the effects of virtual reality technology on various aspects of motor function.

### 2.2 Data source

The search timeframe ranged from the inception of the database to 6 December 2024. A comprehensive search was conducted across Chinese databases, namely CNKI, Wanfang Data, SINOMED, and Chinese Medical Journal Full-Text Database, as well as foreign language databases, namely, PubMed, Web of Science, Embase, CINAHL, and Wiley.

### 2.3 Search terms

The Chinese search terms were as follows: “virtual reality technology/immersive gaming/VR/X-box/Kinect/Nintendo,” “critically ill patient/critical intensive care/ICU,” and “exercise/activity/rehabilitation/training.” The English search terms were as follows: “Virtual Reality/VR/immersive multimedia/exergam*/Wii/X-box/Kinect” and “Intensive Care Unit/ICU/Critical Ill*/Critical Car*/Intensive Car*.”

### 2.4 Database search strategy

The search used Medical Subject Headings (MeSH) subject terms/free words, Boolean logic operators, and literature-tracing pathways.

**Figure 1:**
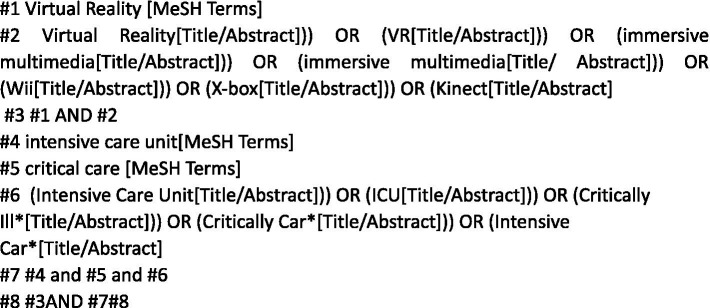
Search strategies for the PubMed database.

Using PubMed as an example, the search strategy is illustrated in Figure 1.

### 2.5 Literature inclusion and exclusion criteria

#### 2.5.1 Inclusion criteria

The inclusion criteria were developed according to the PICOS principles and included the following: ① The study population (P) consisted of critically ill adult patients. ② The intervention (I) was a description of the intervention elements involving VR technology. ③ The comparison measure (C) was another conventional rehabilitation technique. ④ The outcomes (O) included balance function, functional independence, motor function, and walking ability. ⑤ The study type (S) was a randomized controlled trial.

#### 2.5.2 Exclusion criteria

The exclusion criteria included the following: (i) the literature not in Chinese or English, (ii) a Jadad scale score of <4, (iii) raw data that could not be analyzed through meta-analysis, (iv) unavailable full texts, and (v) duplicate publications.

### 2.6 Data extraction

The titles of the retrieved literature were imported to the EndNoteX9, and two researchers independently screened, extracted, and cross-checked the information. If there was disagreement, a third researcher was consulted. The basic information of the extracted RCT literature included the author, country, year, sample, disease types, disease types, intervention measures, VR frequency and cycle, exercise position. For the studies with insufficient information, the original authors were contacted via email or telephone to obtain the missing details.

### 2.7 Literature quality assessment

The latest revised version of the Cochrane-recommended risk of bias assessment tool (ROB2.0) was used by the two investigators to assess the following biases: (1) selection bias, (2) reporting bias, (3) implementation bias, (4) detection bias, (5) loss-to-follow up bias, and (6) other biases. The literature was considered to be at high risk of bias if the study contained one high-risk domain and at low risk of bias if all domains were assessed as low risk. The risk of bias was deemed unknown for the studies evaluated as unclear. The quality of the included literature was assessed using the revised Jadad scale scores, with scores of 1–3 categorized as low-quality literature and scores of 4–7 as high-quality. In cases of disagreement, the third researcher was consulted for adjudication.

### 2.8 Statistical processing

RevMan 5.4 was used for the analysis. After conducting the heterogeneity test, a fixed-effects model was selected if the *p*-value was ≥0.1 and I^2^ was ≤50%. Otherwise, a random-effects model was selected, and sensitivity or subgroup analysis was performed if necessary. The outcome measures included in this study were the Berg Balance Scale, Functional Independence Scale, Fugl-Meyer Assessment scale, and Functional ambulation category, all of which were continuous variables. The standardized mean difference (SMD) or weighted mean difference (WMD) was used as the effect value, and the effect size was expressed as a 95% confidence interval.

## 3 Results

### 3.1 Literature search and process results

**Figure 2:**
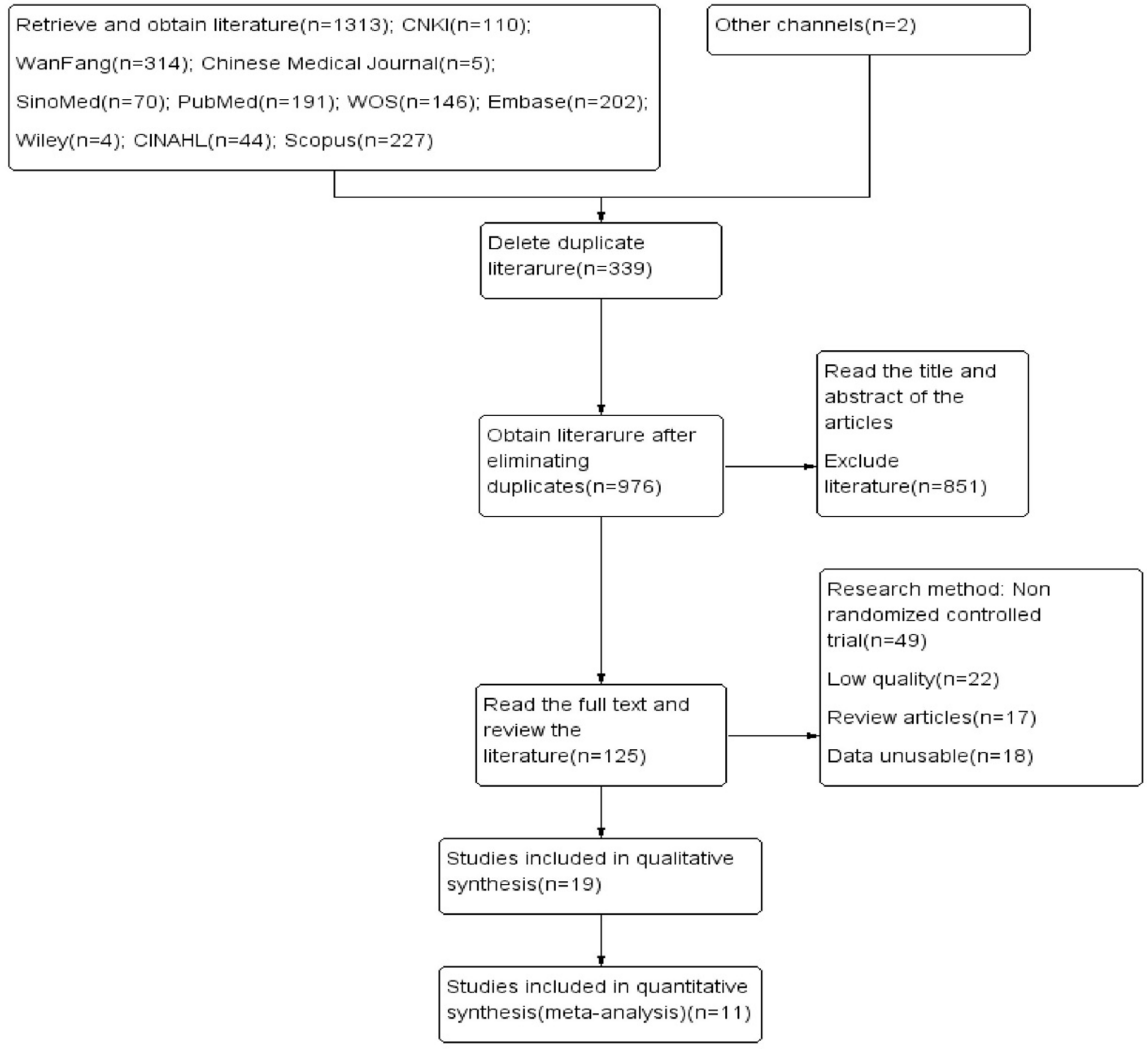
Literature screening process.

The initial search yielded 1,313 articles, and two additional articles were obtained through literature tracing, bringing the total to 1,315 articles. These articles were screened step-by-step, leading to the final inclusion of 11 articles (19–29) in the study. Among them, 5 articles were from mainland China (24–28) and 1 each was from Taiwan (29), Italy (20, 23), Canada (22), Norway (19), and Singapore (21). The detailed literature screening process is shown in Figure 2, and the basic characteristics of the included studies are presented in Table 1.

**Table 1:** Basic characteristics of the randomized controlled trials included.

Author/ Year	Country	Sample	Disease types	Intervention measures	VR frequency and cycle	Exercise position	Outcome measures
		T/C					
Castelli (23), 2023	Italy	12/12	ICU-AW	Immersive VR cycling sports	20-40 min/once per day, 5 days/week, for 3 weeks	Lying→Semi-Fowler’s	④
Gianola (20), 2020	Italy	35/39	TKA	Immersive VR climbing stairs/walking	60 min/once per day, for 1 week	Lying→Semi-Fowler’s → Standing	②
Brunner (19), 2017	Norway	25/27	Stroke	Immersive VR upper and lower limb movements	60 min/once per day, 4–5 times/week, for 4 weeks	Lying→Semi-Fowler’s	②
Saposnik (22), 2016	Canada	47/54	Stroke	Non-immersive VR	60 min/once per day, 10 times in 2 weeks, for 4 weeks	Lying→Semi-Fowler’s	②
Kong (21), 2016	Singapore	31/33	Stroke	Immersive VR boxing/bowling	60 min/once per day, 4 times/week, for 3 weeks	Semi-Fowler’s → Standing	②③
Zhu Haoyuan (24), 2023	China	30/30	Liver transplantation	Immersive VR penguins /remote controlled racing	Complete the corresponding modules per day for 2 weeks	Lying→Semi-Fowler’s → Standing	①
Zeng Yingying (28), 2021	China	38/38	Brain injury	Immersive VR coconut/assembly	10-20 min/once per day, 2–3 times/per week, for 2 months	Semi-Fowler’s → Standing	①③
Mo Yuzhu (27), 2021	China	46/46	Multiple fractures	Immersive VR running/dodging	30–45 min/once per day, 5 times/week, for 3 months	Semi-Fowler’s → Standing	③④
Du Yuanyuan (26), 2023	China	55/55	Stroke	Immersive VR mushroom-picking/racing	30 min/ once per day, 5 days/per week, for 4 weeks	Semi-Fowler’s → Standing	①④
Li Hongmei (25), 2023	China	90/90	Stroke	Immersive VR table tennis/shooting	30 min/once per day, 3 times/per week, for 8 weeks	Lying→Semi-Fowler’s → Standing	①③
Lee (29), 2017	China -Taiwan	26/21	Stroke	Immersive VR meditation	45 min/once per day, twice/per week, for 6 weeks	Lying→Semi-Fowler’s → Standing	①

T represents the experimental group, C represents the control group; ① BBS, Berg balance scale; ② FIM, Functional Independence measure; ③ FMA, Fugl-Meyer Assessment; ④ FAC, Functional ambulation category. “→” represents “transition to.”

### 3.2 Risk of literature bias and quality assessment

**Figure 3:**
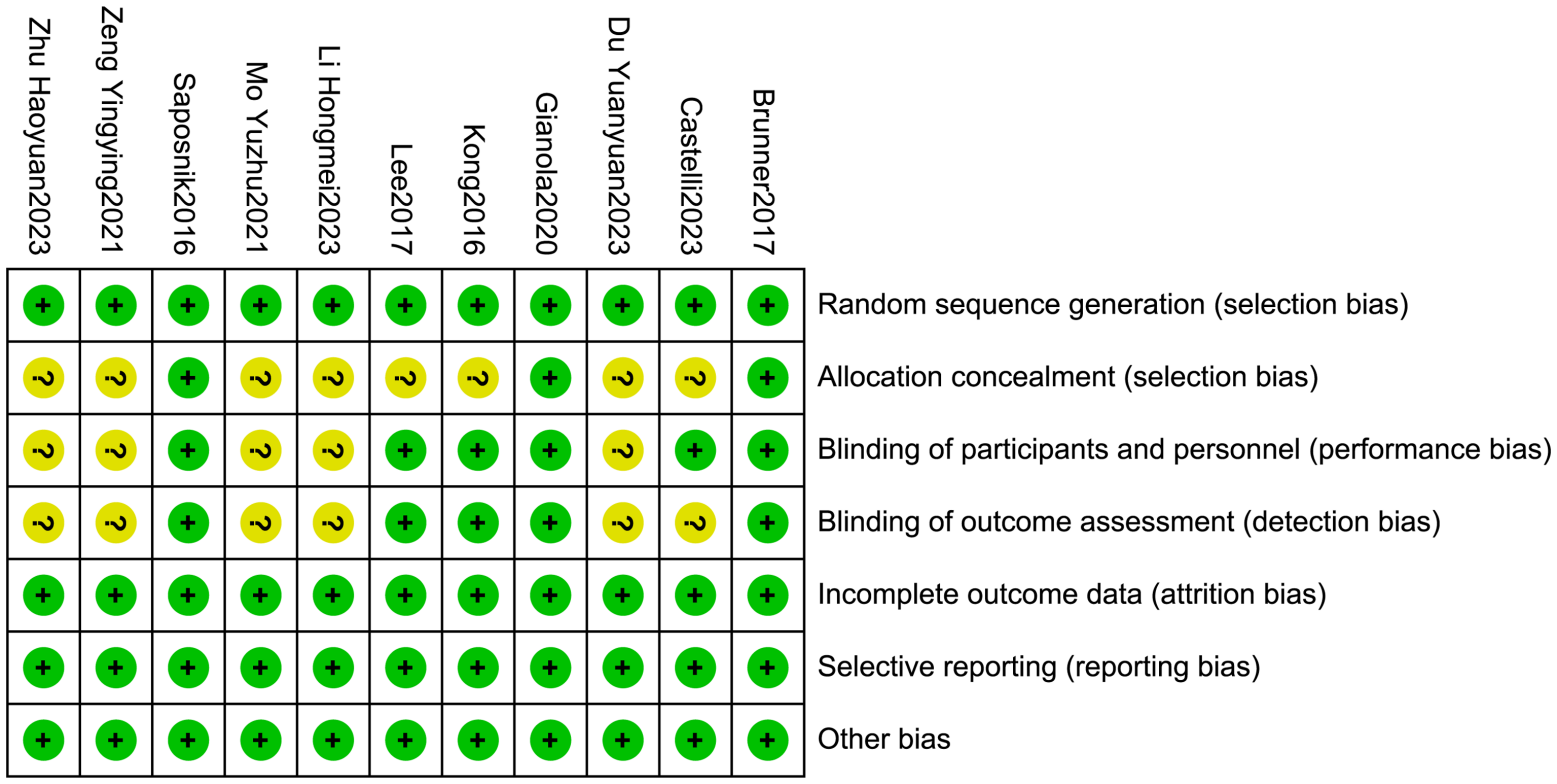
Summary of the risk of bias of the included studies. “+” indicates low risk, and “?” indicates unknown risk.

A total of 11 articles were included in this study (19–29), and randomized grouping was conducted. Among these 11 articles, three (19, 20, 22) clearly explained the random allocation concealment method, while the remaining (21, 23–29) did not provide this information and were rated as unclear. The researchers were not blinded in any of the included studies. A total of 6 studies (19–23, 29) clearly explained that they were single-blinded to the patients. After group discussion, it was unanimously agreed that using blinding on patients would not affect the results and rated them as low risk. The remaining (23–28) did not specify whether blinding was implemented and were rated as unclear. For loss-to-follow up bias, all the inclusion of the studies did not result in the loss of outcome data or the reason for the loss of outcome data could not be related to the true outcome. Therefore, it was unlikely to cause bias and was rated as low-risk. All included studies were at a low risk of selective reporting or other biases. The assessed risk of bias is shown in Figure 3, and the other evaluated quality of the literature is presented in the Supplementary Table 2.

### 3.3 Meta-analysis results

#### 3.3.1 Differences in the BBS scores between the groups

**Figure 4:**
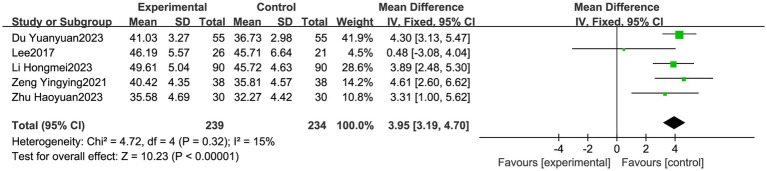
Meta forest plot comparing the Berg Balance Scale scores between the experimental and control groups. VR was superior in improving the BBS scores in the experimental group compared to the control group (*p* < 0.05).

A total of five articles were included (24–26, 28, 29), with 473 patients rated for balance using the BBS—239 in the experimental group and 234 in the control group. The heterogeneity test showed *I*^2^ = 15% and a *p*-value >0.05, indicating little heterogeneity among the studies, so a fixed-effects model was used for the analysis. The results showed that the BBS scores of the experimental group were significantly higher than those of the control group (MD = 3.95, 95% CI: 3.19, 4.70, *p* < 0.05) (Figure 4).

#### 3.3.2 Differences in the FIM scores between the groups

**Figure 5:**
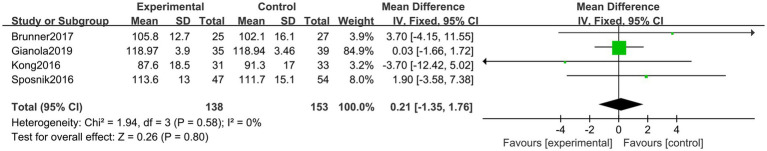
Meta forest plot comparing the functional independence measure scores between the experimental and control groups. VR was not significant in improving the FIM scores (*p* = 0.80 > 0.05).

A total of four articles were included (19–22), with 291 patients rated for functional independence using the FIM—138 in the experimental group and 153 in the control group. The heterogeneity test showed *I*^2^ = 0% and a *p*-value of>0.05, indicating little heterogeneity among the studies, so a fixed-effects model was used for the analysis. The results showed a non-significant effect on the FIM scores between the experimental and control groups (MD = 0.21, 95% CI: −1.35, 1.76, *p* = 0.80, *p* > 0.05) (Figure 5).

#### 3.3.3 Differences in the FAC scores between the groups

**Figure 6:**
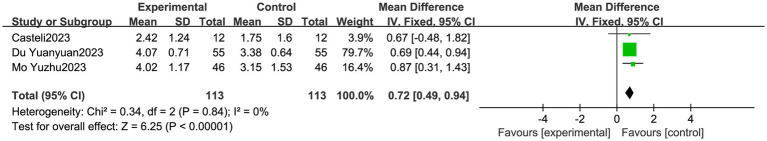
Meta forest plot comparing the functional ambulation category scores between the experimental and control groups. VR was significantly better in improving the FAC scores (*p* < 0.05) in the experimental group compared to the control group.

A total of three articles were included (23, 26, 27), with 226 patients rated for functional walking ability using the FAC—113 in the experimental group and 113 in the control group. The heterogeneity test showed *I*^2^ = 0% and a *p*-value of >0.05, indicating little heterogeneity among the studies, so a fixed-effects model was used for the analysis. The results showed a non-significant effect on the FIM scores between the experimental and control groups (MD = 0.72, 95% CI: 0.49, 0.94, *p* < 0.05) (Figure 6).

#### 3.3.4 The FMA scale for each group

**Figure 7:**
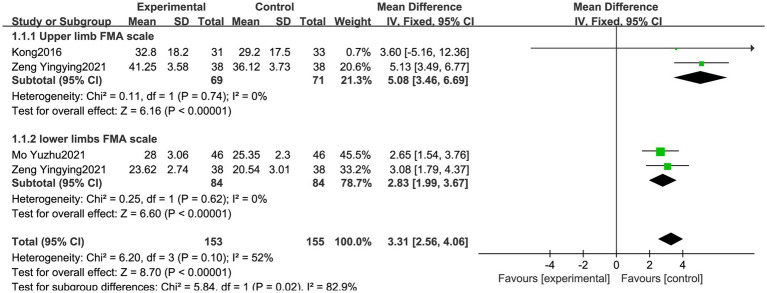
Meta forest plot comparing the Fugl-Meyer assessment scores between the experimental and control groups. VR was statistically significant in improving the upper and lower extremity FMA scores (*p* < 0.05).

A total of three articles were included (21, 27, 28), and the FMA scale scores were analyzed in the subgroups according to the upper and lower limb functions. Upper limb motor function was assessed using the FMA, with 69 cases in the experimental group and 71 cases in the control group. The heterogeneity test showed *I*^2^ = 0% and a *p*-value of >0.05, indicating little heterogeneity across the studies, so a fixed-effects model was used for the analysis. Lower limb motor function was assessed, with 84 cases in the experimental group and 84 cases in the control group. The heterogeneity test showed *I*^2^ = 0% and a *p*-value of >0.05, indicating little heterogeneity across the studies, so a fixed-effects model was used for the analysis. The upper limb FMA scores (MD = 5.08, 95% CI: 3.46, 6.69, *p* < 0.05) and lower limb FMA scores (MD = 2.83, 95% CI: 1.99, 3.67, *p* < 0.05) were significant in both the experimental and control groups, as shown in Figure 7.

#### 3.3.5 Sensitivity analysis

**Figure 8:**
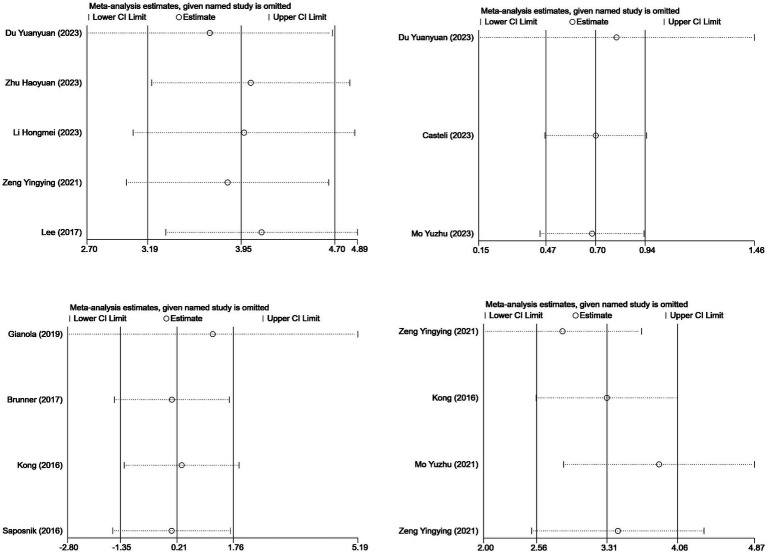
Sensitivity analysis of the outcome indicators for the included studies.

Sensitivity analysis was performed on the outcome indicators using StataMP15, with the method of deleting them one by one, as shown in Figure 8. The sensitivity analysis of the BBS, FIM, FAC, and FMA scores showed that after removing each study and re-merging the analysis, the 95% confidence intervals corresponding to each effect magnitude did not include 0. This indicates that the merged effect significantly deviated from 0, suggesting a significant difference in the mean difference between the experimental group and the control group. After excluding each study, the remaining merged results remained statistically significant and were consistent with the original merged results, indicating the robustness of the results.

#### 3.3.6 Publication bias analysis

Usually, a funnel plot can be used to evaluate publication bias for scales with 10 or more items. As each scale contained fewer than 10 studies, the funnel plot was not clearly displayed. Therefore, Egger’s test was used to report specific *p*-values. The p-values of the Egger’s test for the BBS, FIM, FAC, and FMA scores were 0.124, 0.768, 0.624, and 0.678, respectively, all of which were greater than 0.05. This indicates that the likelihood of publication bias or the possibility of a small sample effect was minimal.

## 4 Discussion

### 4.1 VR technology and conventional traditional rehabilitation techniques

Previous studies (11, 30, 31) have shown the feasibility of early activity in critically ill patients. The control group used traditional rehabilitation techniques, including simple entertainment behavior training (22, 28, 29), active and passive joint activities (20, 21, 23–28), traditional balance training (24–26), coarse movements and flexibility exercises (19), muscle-strengthening training (21, 24, 26), and multidisciplinary rehabilitation nursing (27). A meta-analysis showed that, compared to traditional rehabilitation techniques, VR effectively improved the patients’ overall motor rehabilitation, with significant effects on the BBS, FAC, and upper and lower limb FMA scales, but not on the FIM scale. The reasons may be as follows: (i) the measurement of the FIM scale included both cognitive and motor components, and the original study did not show a specific distribution of scores, making it difficult to determine whether the cognitive component had a low score, which could have resulted in a low overall FIM score; (ii) there was a lack of VR exercises specifically designed for early activity in critically ill patients, which could not meet personalized needs and improve patient functional independence; and (iii) it is also possible that the total intervention time selected for the VR exercise in the original study was insufficient to achieve independent improvement in physical function, ultimately leading to poor results on the FIM scale for some patients. It is suggested that intervention studies be conducted in the future to further explore this and that large-sample research be carried out in the above-mentioned situations.

### 4.2 Research limitations

(1) The strict inclusion and exclusion criteria might have resulted in the incomplete inclusion of the literature and specific selection biases, such as the exclusion of the literature not in Chinese or English. High-quality studies published in other languages were not included, which might have led to selection bias. (2) The intervention time and follow-up duration for each research report were different; in physical rehabilitation training, VR technology lacks unified standards and guidelines, and the intervention duration reported in the 11 studies was inconsistent. Although the duration of each intervention was approximately 45 min and the frequency was approximately three times a week, it might have still led to measurement bias. (3) The limited inclusion of the literature on each outcome measure might have led to small-sample events and publication bias. (4) The research participants did not have the same disease, although they were all critically ill with muscle or motor dysfunction, which might have led to selection bias.

### 4.3 Applicability and implications for the future

Previous studies (32–38) have demonstrated the feasibility of applying VR in critically ill patients. Ma-Huiying et al. (36) reported that VR technology could significantly reduce levels of anxiety, depression, and PTSD in critically ill patients, alleviate their pain perception, and improve their quality of life. There is still a lack of research on the impact of VR on the physiological and motor functions of critically ill patients. Piva et al. (39) studied the early use of VR in critically ill children under the age of 18 years and concluded that early activity programs should be based on individualized interventions, depending on each child’s developmental status. VR bicycle ergometers are feasible and safe, and interdisciplinary collaboration may be conducive to demonstrating the effectiveness of this intervention. Kanschik et al. (40) focused on the overall application of augmented reality (AR) and VR in intensive care medicine and concluded that both VR and AR provide multiple possibilities for improving ICU ward care, whether from the perspective of healthcare professionals or patients. VR and AR will continue to be developed, and their applications in healthcare will expand. Zou et al. (41) mainly studied the application of VR to the mental health, quality of life, and patient satisfaction of critically ill patients. They believed that VR has an impact on the mental health of patients in the intensive care unit (ICU), especially regarding issues related to mental wellbeing and quality of life (QOL). However, its impact on patient satisfaction remains unclear. The abovementioned studies were the latest domestic and international research on VR in critically ill patients. Our study differs from these studies in that it focused on the improvement of motor function in critically ill adult patients using VR, and we drew the following conclusions and implications. Among the 11 studies included in our meta-analysis, the VR game content related to sports rehabilitation included snowball rolling, fruit cutting, coconut picking, bowling, boxing, dodging, table tennis, and shooting. However, none of these activities was specifically designed or strictly controlled for specific diseases or game matching. Therefore, future research can further refine the subgroup analysis for different diseases, upper or lower limbs, fine or gross motor functions, muscle strength, and joint activity. In addition, determining the optimal gaming time—that is, the ideal duration for functional exercise while avoiding adverse experiences such as motion sickness—can help design VR exercise plans in the future. The exercise time mentioned in the 11 studies ranged from three to five times a week, with each session lasting 30–60 min, and no serious adverse reactions were reported, which provides a valuable reference for setting the optimal duration. Finally, a network meta-analysis can be employed in the future to compare whether VR is superior to other forms of physical therapy in the ICU environment, such as acupoint electrical stimulation, biofeedback electrical stimulation, and exoskeleton robots. For now, it is clear that VR can enable critically ill adult patients to engage in and promote active functional exercises of their limbs in a lying or semi-lying position, all while increasing fun and entertainment, thereby surpassing traditional passive movements.

## 5 Summary

The results of this study showed that, compared to traditional rehabilitation care, VR technology has clear advantages for the overall motor function of critically ill patients, providing an evidence-based foundation for applying VR technology to motor function training in critically ill patients. However, there are several limitations, such as the limited number of included studies; differences in research design methods both domestically and internationally; the use of different VR versions and game modules; and the lack of uniform standards for intervention time, intervention frequency, and assessment time selection. The use of VR technology in the rehabilitation of critically ill patients in China is in a stage of rapid development, and it is recommended that we continue to explore specific intervention programs and forms of VR that can be combined with conventional rehabilitation (42). The future requires high-quality, multi-center, and comprehensive rehabilitation programs. High-quality, multicenter, large-scale RCTs are needed to validate this conclusion, and decision-makers need to comprehensively assess exercise function to select the appropriate VR technology when formulating exercise prescriptions, providing a basis for the standardization of the use of VR technology.

## Data Availability

The original contributions presented in the study are included in the article/Supplementary material, further inquiries can be directed to the corresponding author.
