# Supplementary material for: Effects of virtual reality technology on early mobility in critically ill adult patients: a systematic review and meta-analysis
Source: Front Neurol. 2025 Feb 5;15:1469079. doi: 10.3389/fneur.2024.1469079 (PMC11837775; doi:10.3389/fneur.2024.1469079)
Supplement: Supplementary file 2 [file Table_1.docx]

| **Section and Topic** | **Item #** | **Checklist item** | **Location where item is reported** |
| --- | --- | --- | --- |
| **TITLE** | | |  |
| Title | 1 | Meta-analysis of the Effectiveness of Virtual Reality Technology on Motor Function Rehabilitation in Adult Critically Ill Patients: a system review |  |
| **ABSTRACT** | | |  |
| Abstract | 2 | Background: VR technology offers a novel approach to the daily exercise rehabilitation of critically ill patients, and it is anticipated that this will soon become mainstream in the field of critical care medicine. However, existing research findings exhibit variations, indicating differing effects of incorporating VR into the exercise function rehabilitation for critically ill patients.  Objective: To explore the effect of virtual reality technology in motor function rehabilitation care for critically ill patients.  Data resource: Chinese databases CNKI, WanFang Database, SinoMed, Chinese Medical Journal Full Text Database, foreign language databases PubMed, Web of Science, Embase, CINAHL, Wiley. the search time limit is from the construction of the database to 8 April 2024.  Inclusion criteria: Developed according to PICOS principles ① The study population (Population) was adult critically ill patients; ② The intervention (Intervention) was a description of the intervention elements involving VR technology; ③ The control measure (Comparison) was other conventional traditional rehabilitation techniques. Outcomes were balance function, functional independence, functional walking, motor function, and functional walking scale. ⑤Study type (Study) was a randomized controlled trial.  Exclusion criteria: (i) non-Chinese and English literature; (ii) Jadad scale score <4; (iii) raw data could not be analyzed by meta-analysis; (iv) full text was not available; (v) duplicate publication.  Participants: Critically ill adult patients.  Intervention measures: The improvement effect of virtual reality technology on patients' motor function.  Research Assessment and Integrative Methodologies: The data types of each scale belong to continuous variables. The mean and standard deviation of each scale are collected separately, and a fixed effects model is selected using the inverse variance method. Meta-analysis was conducted on different scales measuring motor function, including the Berg balance scale (BBS), Functional independence measure (FIM), Fugl-Meyer Assessment (FMA), and Functional ambulation category (FAC) scales, to understand the effects of virtual reality technology on different aspects of motor function.  Results: 11 RCTs published over 10 years were included, with 880 adult critically ill patients, 435 in the experimental group and 445 in the control group. Meta-analysis showed that compared with the control group, virtual reality technology significantly improved the Berg Balance Scale (BBS) scores of adult critically ill patients (MD = 3.95, 95% CI: 3.19, 4.70, P < 0.05), Functional Independence Measure (FIM) score (MD = 0.21, 95% CI: -1.35, 1.76, P > 0.5), Functional Ambulation Category (FAC) score (MD=0.72, 95% CI: 0.49, 0.94, P<0.05), upper limb motor function (Fugl-Meyer Assessment, FMA) score (MD=5.08, 95% CI: 3.46, 6.69, P<0.05), lower limb motor function (Fugl-Meyer Assessment FMA) scores (MD = 2.83, 95% CI: 1.99, 3.67, P < 0.05).  Conclusion: Compared with traditional conventional rehabilitation techniques, virtual reality technology has a better overall effect in improving motor rehabilitation of critically ill patients, which can improve balance, functional walking, and upper and lower limb motor function of critically ill patients, but the effect of enhancing functional independence of limbs is not yet evident, and it still needs to be further confirmed by high-quality, multi-center, and large-sample clinical trial studies.  Prospero register No.: CRD42024546409. |  |
| **INTRODUCTION** | | |  |
| Rationale | 3 | Several scoping reviews of virtual reality technology have explained the application of virtual reality technology in critical patients' movement, pain, sleep, cognition, anxiety, depression, or other qualitative research. The conclusions regarding the improvement of exercise in the original research are inconsistent. There is currently no quantitative meta-analysis on different aspects of motor function in critically ill patients. |  |
| Objectives | 4 | PICOS: ① Population: Adult critically ill patients; ② (Intervention): Description of intervention elements involving VR technology; ③ (Comparison): Other conventional traditional rehabilitation techniques. ④ (Outcomes): Balance function, functional independence, functional walking, motor function, functional walking scale. ⑤ (Study): It is a randomized controlled trial. |  |
| **METHODS** | | |  |
| Eligibility criteria | 5 | The plan has been registered on PROSPERO([PROSPERO (york.ac.uk)](https://www.crd.york.ac.uk/PROSPERO/)), registration No.: CRD42024546409. |  |
| Information sources | 6 | Chinese databases CNKI, WanFang Database, SinoMed, Chinese Medical Journal Full Text Database, foreign language databases PubMed, Web of Science, Embase, CINAHL, Wiley. the search time limit is from the construction of the database to 8 April 2024. |  |
| Search strategy | 7 | The search used MeSH subject terms/free words, Boolean logic operators, and literature tracing pathways. |  |
| Selection process | 8 | The initial search yielded 1313 documents, and 2 documents were obtained from the literature tracing route for a total of 1315 documents, which were screened step by step for the final inclusion of 11 literature into the study. Among them, 5 articles were from mainland China, 1 from Taiwan, Italy 2 articles, Canada 1 article, Norway 1 article, Singapore 1 article. |  |
| Data collection process | 9 | Extract basic information of eligible randomized controlled trials, including author, country, year, sample size, intervention method, intervention duration, and outcome indicators, as a general data feature table of the literature. The extracted outcome indicators are mainly data from various exercise-related scales, and the data type is continuous data. Extract the mean and standard deviation, sample size of the experimental and control groups for meta-analysis. For those that cannot be directly extracted, convert the data into mean and standard deviation before conducting meta-analysis. Add literature with insufficient information to the original author via email or phone. |  |
| Data items | 10a | Conduct a meta-analysis of different scales for measuring motor function, including the Berg Balance Scale (BBS), Functional Independence Measure (FIM), Fugl Meyer Assessment (FMA), and Functional Walking Category (FAC) scale. These studies are continuous variables; The standardized mean difference (SMD) or weighted mean difference (WMD) is used as the effect value, and the inverse variance method is used. The effect size is expressed as a 95% confidence interval. The intervention time is about 1-3 months, 5 times a week for about 30 minutes each time, and the changes in the scale values before and after the intervention are measured. The included studies have relatively uniform intervention times and cannot achieve absolute consistency, which is also one of the limitations of this article. According to the rehabilitation training guidelines, the intervention time should not be too short, otherwise it will not be included. |  |
|  | 10b | The basic information of the extracted RCT literature included author, country, year, sample size, intervention, intervention duration, and outcome indicators, and those with insufficient information were added together with the original authors by email or telephone. The funding comes from the corresponding author's provincial-level project: Design and Application of a Virtual Reality System for Critical Care Nursing Technology Training. Prospero register No.: CRD42024546409. |  |
| Study risk of bias assessment | 11 | The latest revised version of the Cochrane Recommended Risk of Bias Assessment Tool, ROB2.0, was used by 2 investigators to assess (1) selection bias, (2) reporting bias, (3) implementation bias, (4) detection bias, (5) loss to visit bias; and (6) other bias. |  |
| Effect measures | 12 | In the included studies, data from relevant scales before and after intervention were collected, and the data type was continuous. In addition, the sample size, mean, and standard deviation of the experimental group and control group were also collected. Combine the effect quantity WMD and use a fixed effects model. |  |
| Synthesis methods | 13a | Literature that meets the above inclusion and exclusion criteria can extract effective interventions with specific intervention duration, frequency, and cycle, and include measurement values of scales before and after intervention, which can be extracted or converted into mean and standard deviation. Literature that meets the inclusion and exclusion criteria mentioned above can extract effective intervention measures with specific intervention duration, frequency, and period, including pre - and post intervention scale measurements that can be extracted or converted into mean and standard deviation. Conduct a meta-analysis and create a forest plot to observe the I value. If the I value is greater than 50%, it can be classified into statistical, clinical, or methodological heterogeneity based on the source of heterogeneity. Sensitivity analysis methods such as random effects model combined with effect size, subgroup analysis, meta regression, and mixed effects model can be used. If the heterogeneity is too large and cannot be resolved, consider abandoning meta-analysis. The heterogeneity of this study is all<50%. |  |
|  | 13b | In the case of missing data, the original author will be contacted, and in the case of inability to contact, it can only be abandoned. For some charts, partial data can be obtained through software conversion, such as Origin software. Some literature may need to calculate standard deviation based on quartiles, ranges, standard errors, etc. The studies included in this article can directly obtain the required data and have not yet addressed the above situations. |  |
|  | 13c | Using Excel spreadsheets, extract basic information of literature that meets the requirements based on research objectives and types, and link Excel and Word to achieve synchronous table management. |  |
|  | 13d | RevMan 5.4 software was used for analysis. After the heterogeneity test, if P ≥ 0.1 and I2 ≤ 50%, a fixed-effects model was selected; otherwise, a random-effects model was selected, and sensitivity analysis or subgroup analysis was performed if necessary. This study was a continuous variable; standardized mean difference (SMD) or weighted mean difference (WMD) was used as the effect value, and the effect sizes were expressed as 95% confidence intervals. Sensitivity analysis for inclusion in publication bias was conducted using Stata MP15. All images in the literature are saved in EPS or PNG format, and then created into images with a resolution of 300 and appropriate size using PS drawing software. |  |
|  | 13e | Heterogeneity assessment mainly involves two aspects. On the one hand, statistical heterogeneity can be evaluated by using a random effects model to combine effect sizes. On the other hand, there is heterogeneity in clinical or methodological aspects, which can be explored by exploring the sources of heterogeneity, conducting subgroup analysis, conducting meta-regression and mixed effects models, and conducting sensitivity analysis. If the heterogeneity is too obvious to be resolved, consider abandoning meta-analysis. Heterogeneity tests can include Q test, I^2^ statistic, other tests such as H statistic, Galbraith plot, L'Abi plot, etc. In this paper, Revman 5.4 software is used to calculate the I^2^ statistic for judgment. This article divides motor function into four different scales that reflect different aspects of motor function, with each scale having an I^2^<50%, indicating low heterogeneity. |  |
|  | 13f | Sensitivity analysis is a stable and reliable analytical method for evaluating meta-analysis results, which refers to changing certain influencing factors such as inclusion criteria, differences in research quality, loss to follow-up, statistical methods, etc. For example, after deleting a large sample study, observing whether the combined effect size changes, in order to determine the robustness and reliability of the results. This article uses StataMP15 to conduct sensitivity analysis on the data. After removing each study one by one, the data are all within the confidence interval, indicating that the Meta results are stable and reliable. |  |
| Reporting bias assessment | 14 | There are three main methods for identifying and handling publication bias: funnel plot method, Egger linear regression method, and pruning method. The most commonly used is the funnel plot, but the most commonly included literature should be at least 10. This study conducted a meta-analysis for each scale separately, with less than 10, and it is not recommended to use it. If the intercept a of Egger's test corresponds to P<0.05 or the 95% CI does not include 0, it indicates publication bias. The P values obtained in this study are all greater than 0.5, indicating a low likelihood of small sample probability or small sample effect. |  |
| Certainty assessment | 15 | In meta-analysis, the methods for evaluating the certainty (or confidence) of the evidence in the results mainly include: first, data extraction and evaluation. To ensure the accuracy of data extraction, it is recommended that at least two researchers independently extract data and use consistency tests (such as Kappa value) to evaluate the consistency of the extracted results. In case of inconsistent extraction results, third-party researchers can be invited to participate in discussions to resolve the differences. Secondly, consider the heterogeneity of data: when extracting data, pay attention to identifying and recording factors that may lead to data heterogeneity, such as patient characteristics, intervention measures, outcome indicators, etc. from different studies. In addition, combining professional knowledge to determine the clinical significance of the results: Meta analysis results should not only consider whether they have statistical significance, but also combine professional knowledge to determine whether the results have clinical significance. If the combined effect size has clinical significance but no statistical significance, it cannot be concluded and further data collection is required. Finally, report writing and adherence to standards: The PRISMA standard can be referenced to write the entire text. The PRISMA standard (Meta Analysis Reporting Standard) is an important reference and reporting standard for conducting meta-analysis, which helps ensure the quality and consistency of the report. Through the above methods, meta-analysis can provide a relatively objective and comprehensive evaluation. |  |
| **RESULTS** | | |  |
| Study selection | 16a | 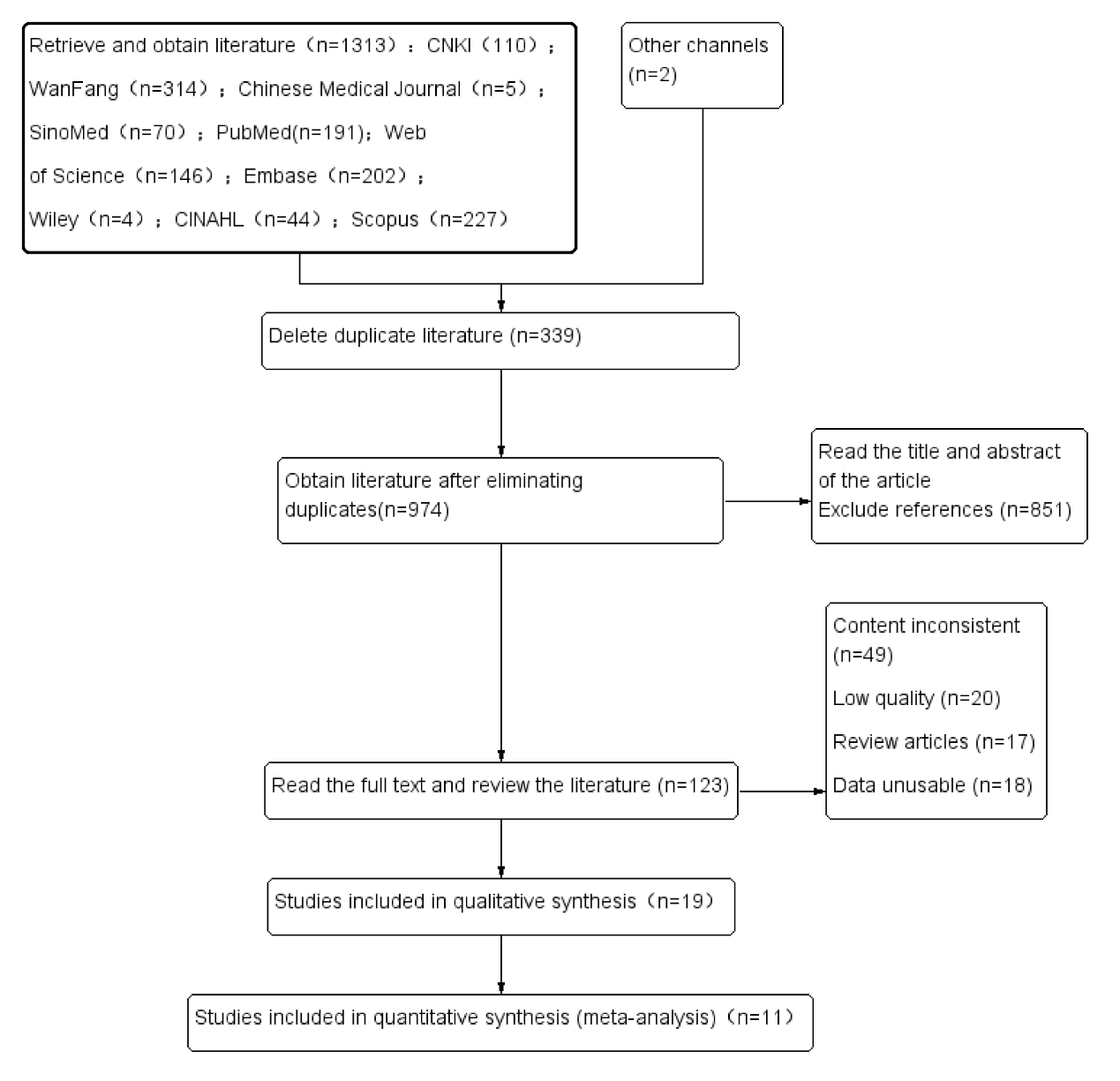 |  |
|  | 16b | There is an article published by Zhao Jiangong in 2023 titled "Analysis of the therapeutic effect of virtual reality technology training on cognitive and motor function rehabilitation after severe traumatic brain injury surgery". Due to the lack of clear indication on whether it is a randomized controlled study, the Jadad score is of low quality and was not included in the literature. There is also an article by Xia Yanling in 2023 on the application of virtual reality technology in early activities of ICU patients, which only includes indicators of grip strength and the number of cases of acquired frailty, which does not meet the data requirements of this study. Others were excluded due to belonging to the category of scheme construction, non critical patients, current situation surveys, review literature, and so on. |  |
| Study characteristics | 17 | 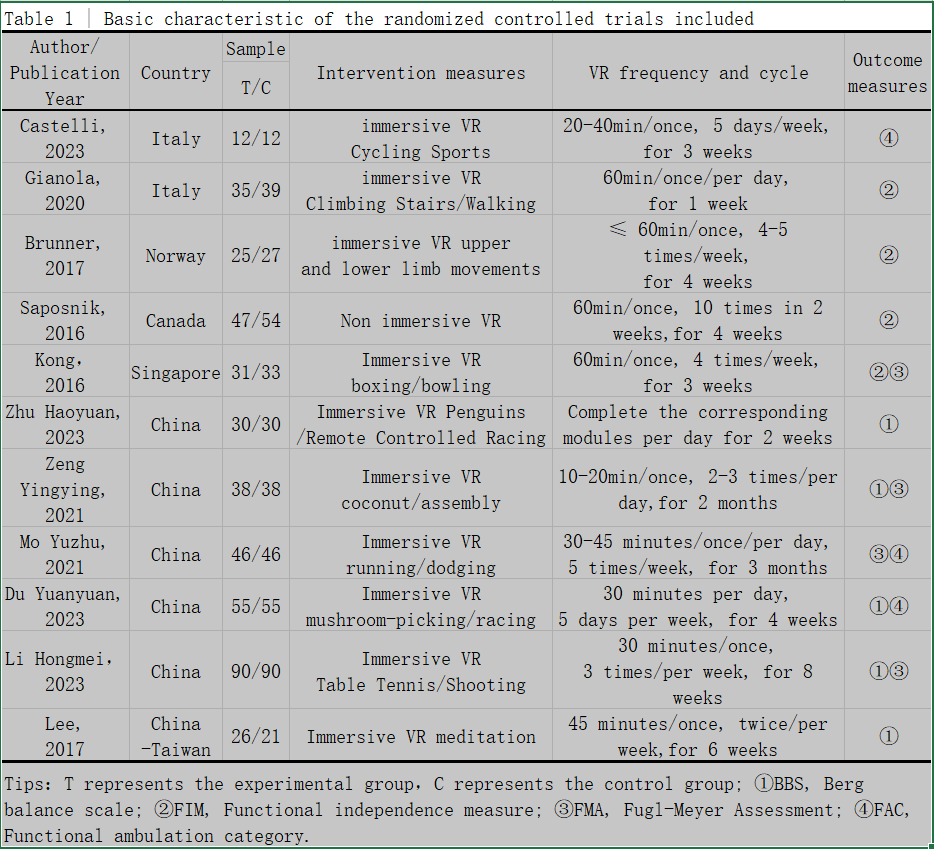 |  |
| Risk of bias in studies | 18 | 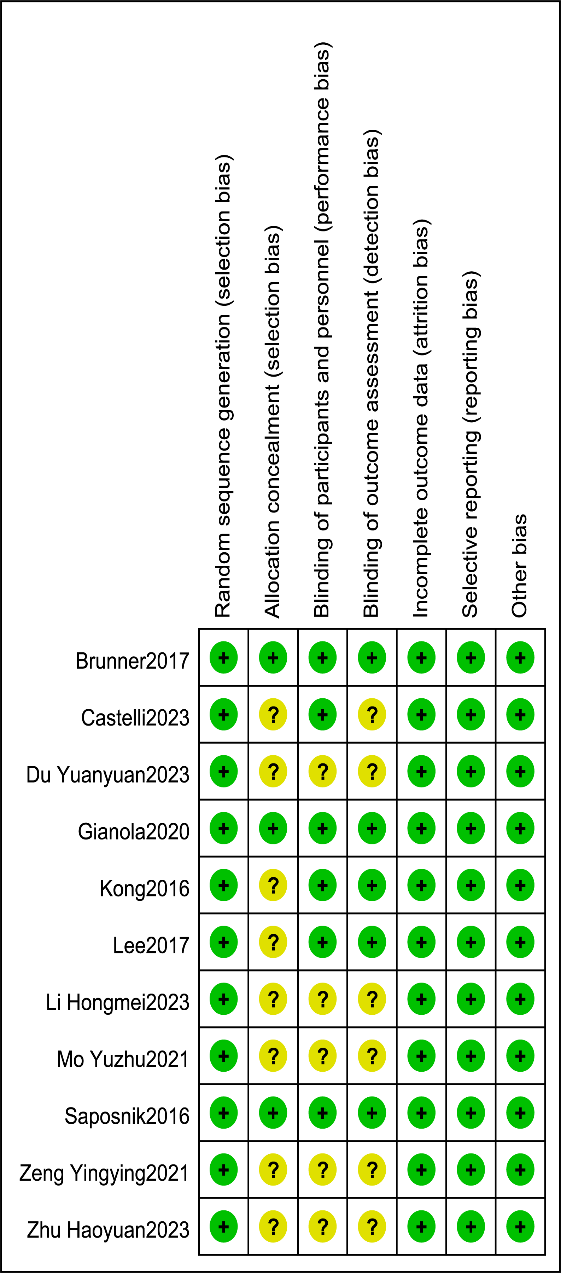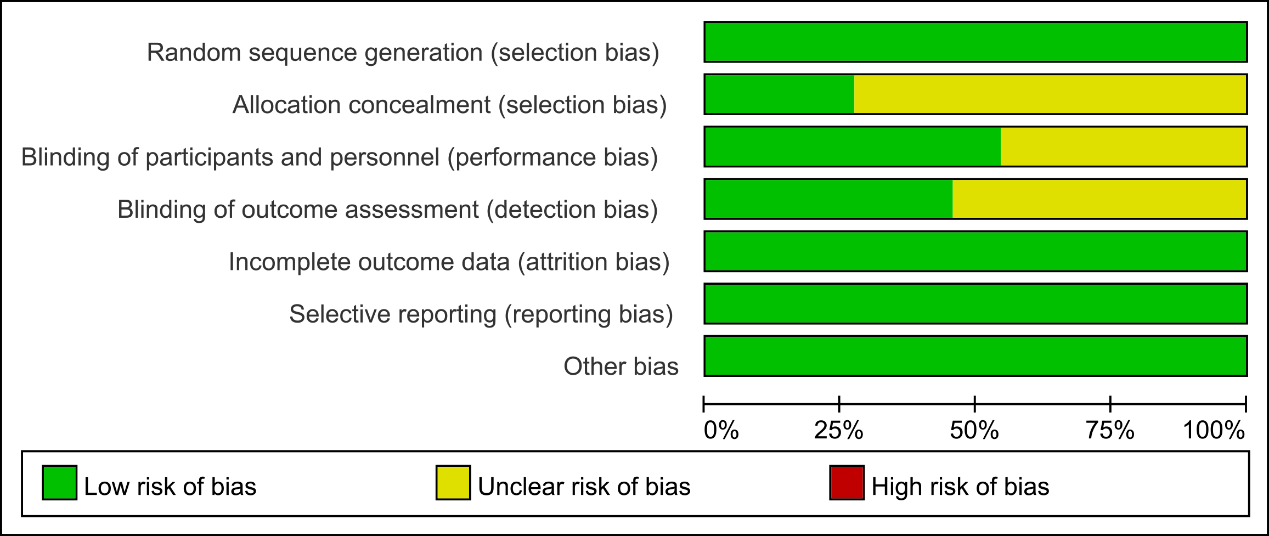 |  |
| Results of individual studies | 19 | 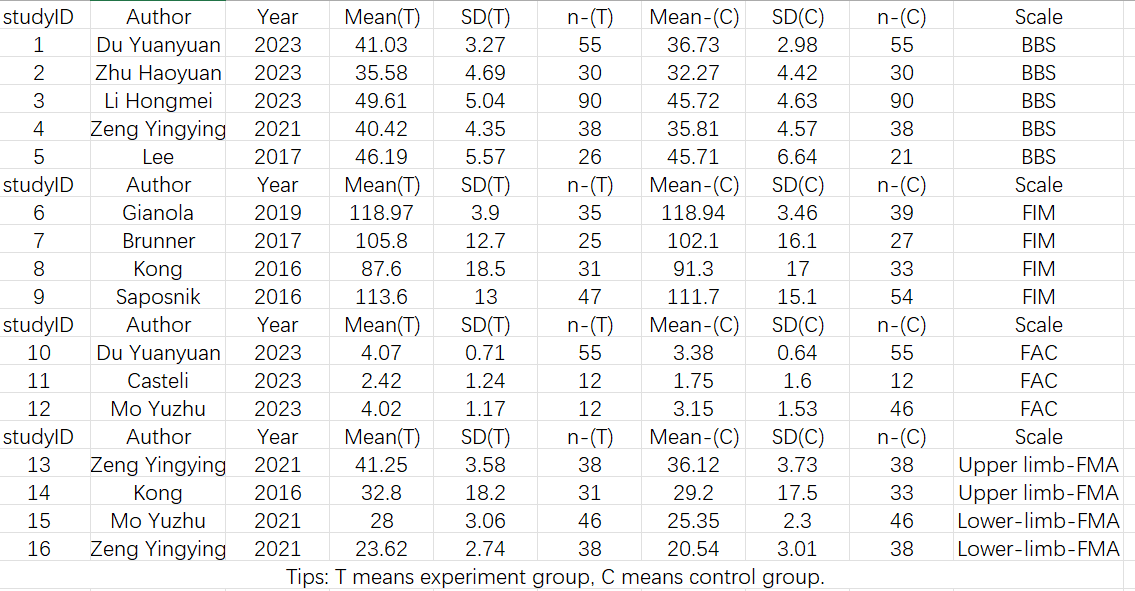 |  |
| Results of syntheses | 20a | Eleven articles were included in this study, which clearly described grouping according to treatment and rated as low risk. 3 clearly explained the random allocation concealment method, and the rest were not mentioned in the original article and were selected as unclear. None of the included studies were able to blind the patients, 6 The literature clearly explains that it is single-blinded, and the group discussed that not blinding the patients did not affect the results and rated it as low-risk, the remaining did not specify whether blinding was implemented and rated as unclear.5 literature detailing the implementation of blinding of outcome testers were low risk, and the remaining were not specified and rated as unclear. For missing visit bias, the included studies all processed incomplete outcome data, which was unlikely to cause prejudice and was rated low risk. |  |
|  | 20b | Differences in BBS scores between groups  A total of 5 articles were included[11-13, 15, 16] literature, 473 patients were rated for balance using BBS, 239 in the experimental group and 234 in the control group.I2 =15%, P>0.05, there was little heterogeneity among the studies, and the fixed-effects model was used for analysis. The results showed that the BBS scores of the experimental group were significantly higher than those of the control group (MD = 3.95, 95% CI: 3.19, 4.70, P < 0.05).  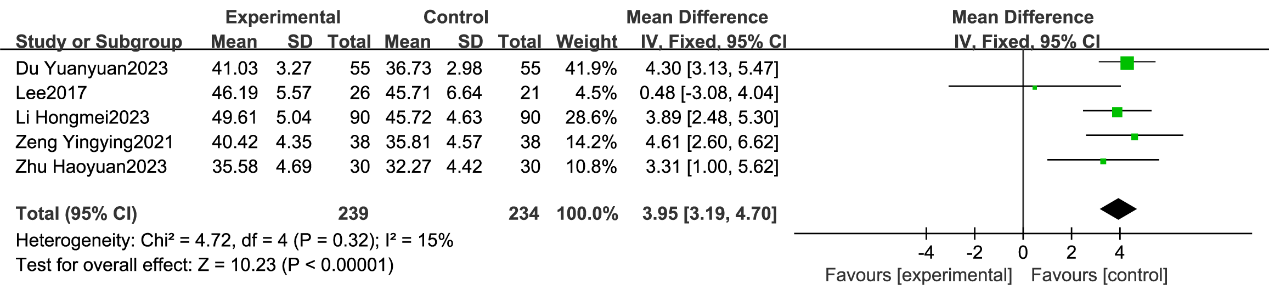  Differences in FIM scores between groups  A total of 4 articles were included[6-9] literature; 291 patients were rated on balance using BBS, 138 in the experimental group and 153 in the control group.I2 =0%, P>0.05, small heterogeneity across studies, analyzed using a fixed effects model. The results showed a non-significant effect on FIM scores in the test and control groups (MD = 0.21, 95% CI: -1.35, 1.76, P > 0.05).  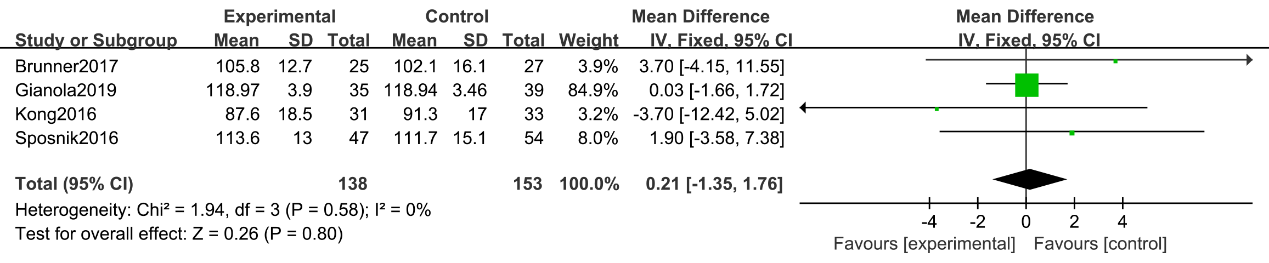  FAC scale for each group  A total of 3 articles were included[10, 13, 14] literature; 226 patients were rated on balance using BBS, 113 in the experimental group and 113 in the control group.I2 = 0%, P > 0.05, small heterogeneity across studies, analyzed using fixed effects model. The results showed a non-significant effect on FIM scores in the test and control groups (MD = 0.72, 95% CI: 0.49, 0.94, P < 0.05).  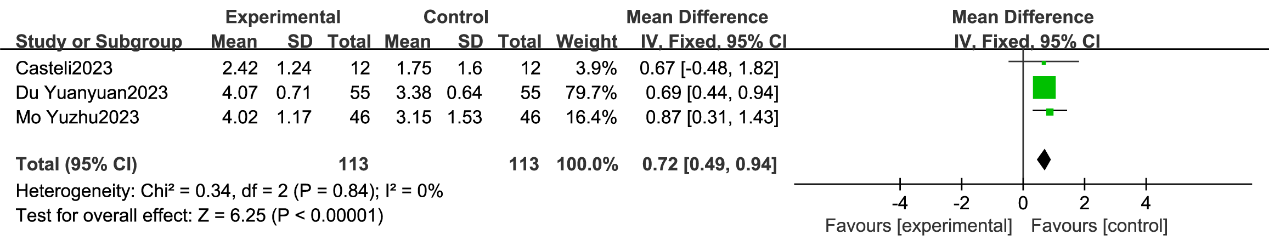  FMA scale for each group  A total of 3 articles were included[8, 14, 15], and FMA scale scores were analyzed in subgroups according to the upper limb and lower limb; upper limb motor function was assessed using FMA, of which 69 cases were in the experimental group and 71 cases in the control group, I2 = 0%, P > 0.05, small heterogeneity across studies, and analyzed using a fixed effects model. Lower limb motor function was assessed, of which 84 cases in the test group and 84 cases in the control group, I2 =0%, P>0.05, with small heterogeneity across studies, and analyzed using a fixed effects model. The results showed that the effect of the experimental and control groups was significant on the upper limb FMA scores (MD = 5.08, 95% CI: 3.46, 6.69, P < 0.05) and the lower limb FMA scores (MD = 2.83, 95% CI: 1.99, 3.67, P < 0.05).  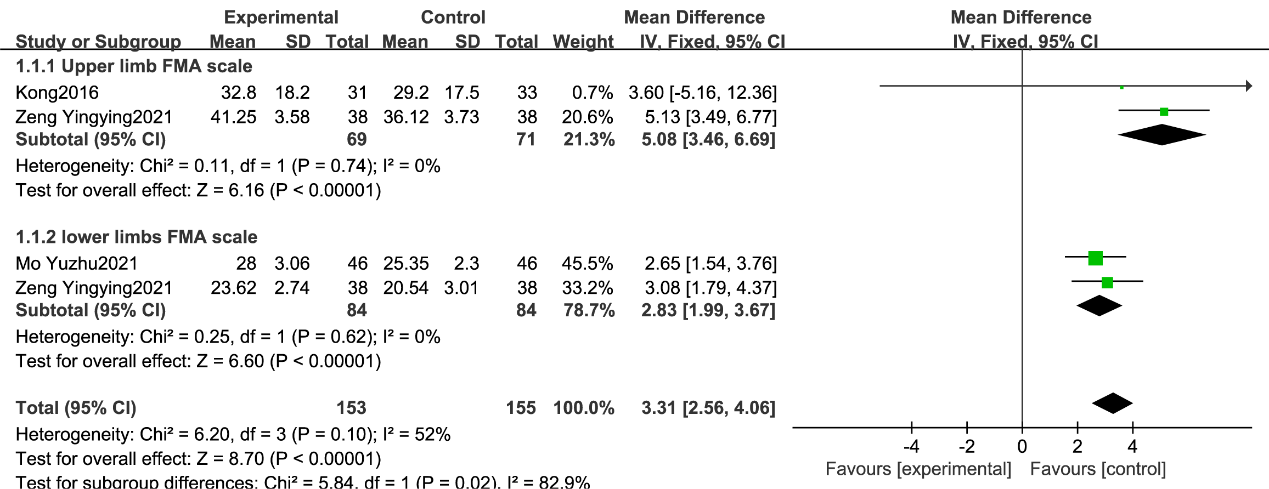 |  |
|  | 20c | This study shows that heterogeneity I^2^ is all<50%, indicating low heterogeneity. If analyzed, heterogeneity caused by meta-methodology is excluded due to using the same scale. The main consideration is that the included studies are of different races, disease types, and virtual reality software versions from different countries. Although the intervention duration is similar, it is not the same. There is also the fact that the population has no previous experience of playing games, which is not mentioned in all the original literature. There is no way to investigate. The above are possible sources of heterogeneity. The heterogeneity is small and within the acceptable range. |  |
|  | 20d | 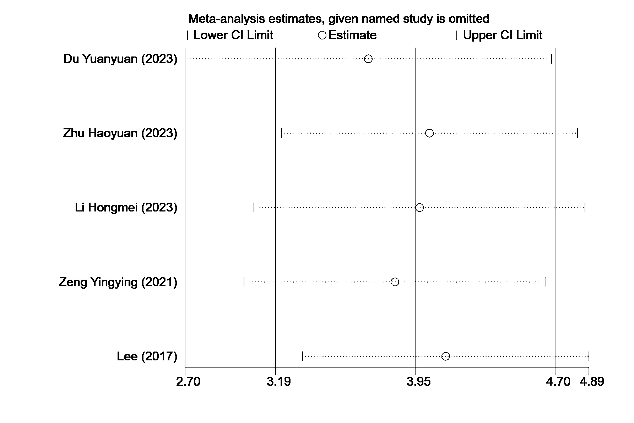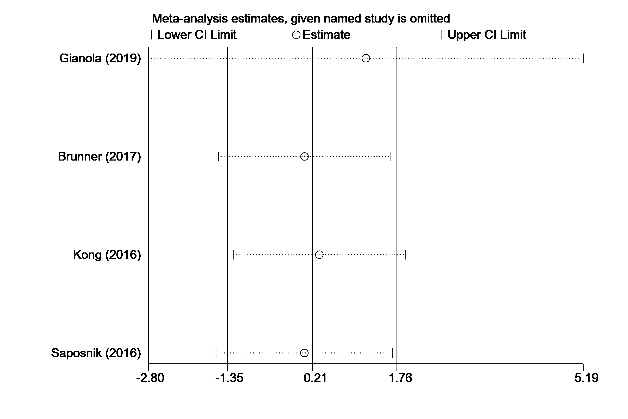  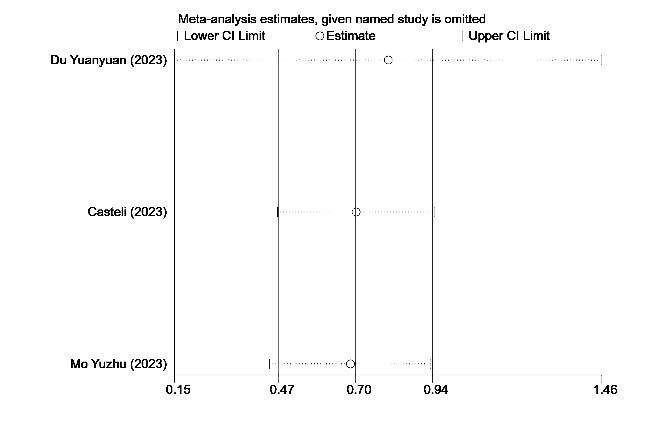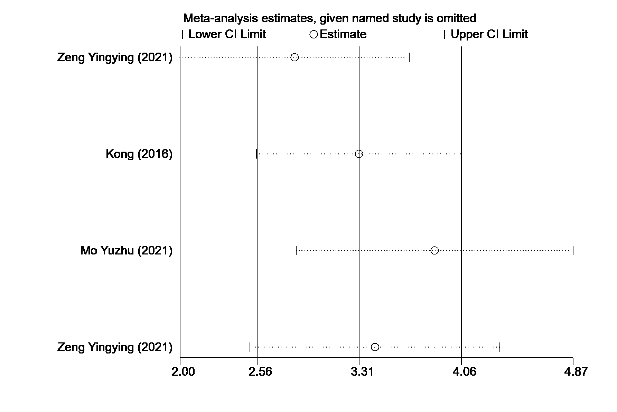 |  |
| Reporting biases | 21 | Because fewer than 10 studies were included in each scale, the funnel plot was not used for publication bias assessment, and the Egger test was used instead. It was found that the p-values of the Egger test for the BBS, FIM, FAC, and FMA scores were 0.124, 0.768, 0.624, and 0.678, respectively, which were all p > 0.05, indicating that the likelihood of publication bias is small or that the possibility of a small sample effect is Small. |  |
| Certainty of evidence | 22 | Certainty (or confidence) of evidence in this study: Firstly, data extraction and evaluation. At least two researchers independently extract data and use consistency tests (such as Kappa values) to evaluate the consistency of the extracted results. When the results are inconsistent, third-party researchers participate in discussions and resolve differences. Secondly, data heterogeneity: When extracting data, identifying and recording factors that may lead to data heterogeneity, such as patient characteristics, intervention measures, outcome indicators, etc. from different studies, are presented in the form of charts to illustrate the basic characteristics of the literature. In addition, combining professional knowledge to determine the clinical significance of the results: the size of the combined effect, the data has statistical significance. Finally, the report writing and adherence to standards: The entire text was written in accordance with PRISMA standards. There are PRISMA checklists and PRISMA literature screening flowcharts as evidence. The above proves that this study can provide a relatively objective and comprehensive evaluation. |  |
| **DISCUSSION** | | |  |
| Discussion | 23a | The results of this study show that compared with traditional rehabilitation care, VR technology has obvious advantages for the overall motor function of critically ill patients, providing an evidence-based basis for applying VR technology to the motor function training of critically ill patients. However, there are some limitations, such as the limited number of included studies, some differences in research design methods at home and abroad, the use of different VR versions and game modules, and the lack of uniform standards for intervention time, intervention frequency, and assessment time selection, etc. The use of VR technology in the rehabilitation of critically ill patients in China is in a stage of rapid development, and it is recommended that we continue to explore the specific intervention programs and forms of VR that can be combined with conventional traditional rehabilitation.The future still needs high-quality, multi-center, and comprehensive rehabilitation programs. High-quality, multicenter, large-sample RCTs are needed to validate this conclusion, and decision-makers need to comprehensively assess the function of exercise to select the appropriate VR technology when formulating exercise prescriptions to provide a basis for further standardization and standardization of the use of VR technology. |  |
|  | 23b | (1) the inclusion and exclusion criteria are strict, which may lead to incomplete inclusion of the literature, and there may be a particular selective bias; (2) the inclusion of the literature in the language limitations, which may omit the high quality of the literature; (3) the inclusion of the literature on each outcome indicator is limited, which may cause a particular risk of bias. (4) The limited number of literature included for each outcome indicator may result in a specific risk of bias. |  |
|  | 23c | (1) The intervention time and follow-up time for each study are different; In physical rehabilitation training, VR technology lacks unified standards and guidelines, and the relationship between intervention time and efficacy cannot be clearly defined; (2) Differences in the population included in the literature cannot be obtained, and previous gaming experience cannot be obtained. |  |
|  | 23d | Practice: Whether to extensively apply virtual reality technology to critically ill patients, and the cost-effectiveness ratio of the application.  Policy: More effective after application, may affect the tilt of policies. For example, in order to better promote early recovery of patients and achieve good therapeutic effects, the government may involve medical insurance to reduce the burden on patients while improving their quality of life.  In the future, there is still a need for multi center, large sample, high-quality randomized controlled trials (RCTs) on virtual reality technology. Randomized controlled trials or network meta-analyses can also be conducted on commonly used rehabilitation techniques, which is more conducive to obtaining the effects of various rehabilitation techniques and providing better references for the government to understand cost-effectiveness ratios. |  |
| **OTHER INFORMATION** | | |  |
| Registration and protocol | 24a | The plan has been registered in PROSPERO, registration No.: CRD42024546409. The registration date is May 27th, and the registered name is: Meta analysis of the effect of virtual reality technology on motor function rehabilitation in adult critically ill patients |  |
|  | 24b | The website address is: [PROSPERO (york.ac.uk)](https://www.crd.york.ac.uk/PROSPERO/) |  |
|  | 24c | There haven't been significant changes. |  |
| Support | 25 | The funding for this paper is a provincial-level project led by the corresponding author, titled "Design and Application of a Virtual Reality System for Critical Care Nursing Technology Training". Project of Sichuan Provincial Department of Science and Technology, Project Number: 2022NSFSC1563. The corresponding author is responsible for: Corresponding author is responsible for: writing - review and editing, formal analysis, funding acquisition. |  |
| Competing interests | 26 | Disclaimer: The author of the comment has no competitive interests. |  |
| Availability of data, code and other materials | 27 | The following data information can be obtained from the first author: template data collection forms; data extracted from included studies; data used for all analyses; analytic code; any other materials used in the review. |  |

*From:*  Page MJ, McKenzie JE, Bossuyt PM, Boutron I, Hoffmann TC, Mulrow CD, et al. The PRISMA 2020 statement: an updated guideline for reporting systematic reviews. BMJ 2021;372:n71. doi: 10.1136/bmj.n71
